# The 9 bp Deletion between the Mitochondrial COII and Lysine tRNA Genes in a Caucasian Population with Cognitive Disorders: An Observational Study

**DOI:** 10.3390/ijms251910826

**Published:** 2024-10-09

**Authors:** Marika Giuliano, Sandro Santa Paola, Eugenia Borgione, Mariangela Lo Giudice, Francesco Domenico Di Blasi, Rosa Pettinato, Corrado Romano, Carmela Scuderi

**Affiliations:** 1 Oasi Research Institute—IRCCS, 94018 Troina, Italy; ssantapaola@oasi.en.it (S.S.P.); eborgione@oasi.en.it (E.B.); mlogiudice@oasi.en.it (M.L.G.); fdiblasi@oasi.en.it (F.D.D.B.); rpettinato@oasi.en.it (R.P.); cromano@oasi.en.it (C.R.); cscuderi@oasi.en.it (C.S.); 2 Section of Clinical Biochemistry and Medical Genetics, Department of Biomedical and Biotechnological Sciences, University of Catania, 95123 Catania, Italy

**Keywords:** mitochondrial DNA, MIC9D, cognitive deficits, neuromuscular disorders, behavioral disorders

## Abstract

The loss of one of the two copies of the 9 bp tandem repeat sequence (CCCCCTCTA) located in the small non-coding region between the cytochrome oxidase II (COII) and the lysine tRNA genes in human mtDNA has been reported to be polymorphic in Asian, Oceanian and Sub-Saharan African populations, but it has rarely been observed in Europe. In this study, we will evaluate the possible association between the MIC9D polymorphism and cognitive disorders. A genetic analysis of unrelated Sicilian patients with cognitive deficits was performed to identify the 9 bp deletion MIC9D polymorphism. The MIC9D polymorphism was found in six patients, whereas this variant was absent in control individuals without cognitive deficits. The patients with the MIC9D polymorphism exhibited more complex clinical presentations; in particular, all had neuromuscular disorders and five also presented with behavioral disorders. The present study suggests a potential association between the MIC9D polymorphism and cognitive impairment with concurrent neuromuscular and behavioral involvement.

## 1. Introduction

Mitochondria are organelles responsible for generating cellular energy, as well as phospholipid and heme synthesis, calcium homeostasis, cell death, and apoptotic activation [1]. They possess their own multicopy circular and double-stranded genome, mitochondrial DNA (mtDNA), that comprises 16,569 base pairs and is inherited maternally.

However, the majority of the mitochondrial proteins are encoded by nuclear genes, while only 13 of the respiratory proteins, all 22 mitochondrial transfer RNAs and 2 ribosomal RNAs are encoded by the mitochondrial genome.

About 1.1 kb of the mtDNA genome comprises non-coding regions. A significant portion of the non-coding regions is represented by the D-loop, which plays a central role in the mtDNA replication and transcription processes. Alongside the D-loop, the non-coding region of the mtDNA is represented by OriL, the origin of replication on the light strand, and the hypervariable segments, the most polymorphic regions of mtDNA. In addition to this, mtDNA contains limited non-coding intergenic sequences and the genes have none or only a few non-coding bases between them [2].

The inheritance from generation to generation of a cluster of single nucleotide polymorphisms (SNPs) in mtDNA differentiates the various haplotypes, which is useful for defining geographic genetic populations. Specifically, the loss of one of the two copies of the 9 bp tandem repeat sequence (CCCCCTCTA), located in the small non-coding segment between the cytochrome oxidase II (COII) and the lysine tRNA genes in human mtDNA, has been extensively used as a genetic marker in phylogenic studies. The 9 bp deletion (MIC9D) has been reported to be polymorphic in Asian, Oceanian and Sub-Saharan African populations, but it has rarely been reported in Europe [3].

Although MIC9D was considered a non-pathogenic geographic polymorphism, it has been reported in various diseases also in Asian populations. In particular, a higher prevalence of MIC9D was found in individuals of Taiwanese families with MELAS (mitochondrial encephalomyopathy with lactic acidosis and stroke-like episodes) and MERRF (myoclonic epilepsy and ragged-red fibers) syndromes compared with healthy subjects [4]. It is plausible that the polymorphism may be cotransmitted with the respective mutations, m.8344A > G for MELAS and m.3243A > G for MERRF, in the Taiwanese population.

In a Chinese family with essential hypertension, instead, MIC9D was found in combination with the m.16189T > C mutation [5].

In a study investigating mtDNA mutations in matched atrial tissues and blood samples, MIC9D was observed in two of four patients with chronic atrial fibrillation [6].

A systemic study investigating the role of the 9 bp insertion–deletion polymorphism in human disease revealed that an insertion was observed more in the group of patients with spinocerebellar ataxias and idiopathic neurological syndrome, whereas a deletion was observed more frequently in those with dilated cardiomyopathy [7].

Moreover, MIC9D was reported in patients with heterogeneous polycystic ovary syndrome disorder at a higher rate compared to healthy controls [8].

The MIC9D polymorphism has also been associated with gestational diabetes mellitus. In particular, five out of six individuals with MIC9D had a family history of Type 2 diabetes mellitus and required insulin therapy [9].

Furthermore, a case–control study conducted on hepatocellular carcinoma patients and healthy controls in the Chinese population showed that the frequencies of MIC9D were significantly higher in cases than in controls [10].

Previously, we found MIC9D in the homoplasmic condition in lymphocytes and muscle of three Sicilian patients with sensorineural hearing loss and encephalomyopathy [11]. Moreover, two of these patients also presented with cognitive deficit and psychiatric disturbances.

Based on these preliminary findings, we wondered if there was an association between the MIC9D polymorphism and cognitive disorders.

We believe that due to its specific location, the MIC9D polymorphism can alter the expression of mtDNA genes that encode oxidative phosphorylation (OXPHOS) subunits leading to an alteration of mitochondrial energy production. As is known, the energy provided by mitochondria is necessary for the optimal functioning of brain cells and neurogenesis [12]. Mitochondrial bioenergetic dysfunction are indicated to be potentially involved in the etiology of intellectual disability-related neurodevelopmental disorders, such as Down syndrome, Rett syndrome, Fragile X syndrome and autism [12,13].

As proof of this, we examined the presence of MIC9D in 154 Sicilian subjects with different cognitive deficits such as intellectual disability, learning and coordination disability, developmental delay and cognitive decline.

## 2. Results

A genetic analysis of 154 unrelated Sicilian patients with cognitive deficits identified the MIC9D polymorphism in 6 of them (3.9%). This variant was not detected in 147 control individuals without cognitive deficits (Table 1). We performed the Fisher exact test, demonstrating that the difference between these frequencies was statistically significant (*p* = 0.03).

Information on the medical history of the patients with cognitive deficits was reviewed and is summarized in Table 2.

From a multidisciplinary approach, we observed that the patients with the MIC9D polymorphism displayed more complex clinical presentations. In particular, all had neuromuscular disorders and five presented with behavioral disorders, too. Hypotonia was the most frequently represented neuromuscular disturbance which was found only in one patient with motor coordination disorder. Among the behavioral disorders, the most frequently exhibited one was oppositionism (4), followed by aggressiveness (3), and, lastly, provocative attitudes (2) and self-harm (2) (Table 3).

We observed differences between the patients with one and two repeats in terms of the frequency distribution of neuromuscular and behavioral disorders (Table 4 and Table 5).

In detail, approximately 83.3% of the cases with a deletion showed behavioral disorders, while such a feature was found only in 18.2% of the patients without a deletion.

On the other hand, all the cases with a deletion presented neuromuscular disorders, in contrast to only 41.2% of the patients without a deletion.

To verify whether these observed differences were statistically significant, we performed a Fisher exact test, demonstrating that the frequencies were significantly different for both behavioral disorders (*p* = 0.0015) and neuromuscular disorders (*p* = 0.0059).

## 3. Discussion

In this study, we explored the possible association of the MIC9D polymorphism with cognitive impairment. For this purpose, we investigated the MIC9D polymorphism in Sicilian unrelated patients with cognitive deficits, and identified it in the homoplasmic condition in six patients; conversely, this polymorphism was absent in the control individuals without cognitive deficits. These observed differences appear to be statistically significant.

The MIC9D polymorphism has been reported in several diseases, including cardiac [6] and neurological syndromes [7], polycystic ovary syndrome [8], gestational diabetes mellitus [9], and cancer [10]. Moreover, it was found more frequently in individuals with mitochondrial diseases [4].

Our data could add cognitive deficits to the clinical disorders associated with the MIC9D polymorphism.

Furthermore, a detailed clinical data analysis of the patients showed more complex clinical presentations in the patients with the MIC9D polymorphism. Particularly, the individuals with the 9 bp deletion presented with behavioral and neuromuscular features more frequently than those without a deletion. These observed differences were shown to be statistically significant.

Previously, we had found MIC9D in the homoplasmic condition in three Sicilian patients with sensorineural hearing loss and encephalomyopathy [11]. In addition, two of them showed moderate intellectual disability and psychiatric disturbances. It is interesting that a subsequent clinical evaluation also pointed out psychiatric disturbances in the third patient.

It is widely known that mtDNA mutations and deletions contribute significantly to the onset and progression of various brain diseases, including neurodegenerative disorders, stroke, cognitive deficits, and psychiatric illnesses such as schizophrenia and bipolar disorder [14]. Previously, we found a homoplasmic mutation within the mitochondrial cysteine tRNA gene in a patient with intellectual disability, epilepsy, quadriplegia, cerebellar and extrapyramidal signs, and muscle atrophy [15]. Recently, we have found mitochondrial dysfunction due to mtDNA variants in subjects with autism spectrum disorders, intellectual disability and clinical features suggestive of mitochondrial disease [16]. We reported a mutation within the mitochondrial Serine tRNA gene in a patient with profound intellectual disability, spastic quadriplegia, myoclonic epilepticus status, sensorineural hearing loss and myopathy [17].

Due to the high density of mitochondria, muscle and nerve cells are highly dependent on oxidative phosphorylation for energy production and extremely sensitive to its defects [18].

Brain development, mainly synaptogenesis, is prone to mitochondrial abnormalities of respiratory function, Ca^2+^ cycling, ROS/RNS production, mechanisms of programmed cell death, and the misbalance between fusion, fission and autophagy [19].

Likely due to its specific location, the MIC9D polymorphism may alter downstream and upstream gene expression, such as that of MTATP6, MTATP 8, MTCO3, and MTCYB, which code for OXPHOS subunits, leading to a decrease in oxidative phosphorylation and altering ATP generation [7].

Interestingly, the analysis of the 9 bp deletion breakpoint regions revealed the presence of G-quadruplex structures [20], which are hypothesized to affect mitochondrial replication, transcription and translation [21]. These alternative motifs were also located adjacent to mtDNA deletions in several genetic diseases like Kearns–Sayre syndrome, Pearson marrow–pancreas syndrome, mitochondrial myopathy, progressive external ophthalmoplegia, etc. [22]. Dahal et al. [20] proposed a mechanism of generation of a 9 bp deletion induced under stress conditions by endonuclease G released into the mitochondrial matrix.

Certainly, the presence of MIC9D alone is not enough to explain the clinical phenotype of our patients. Therefore, it is plausible that additional factors, such as exposure to environmental noxious agents, secondary mutations or modifying nuclear factors, may play a role. We did not reveal any exposure to toxic factors in the clinical history of our patients, but we cannot exclude pre- or perinatal exposure. Moreover, our patients with MIC9D underwent genetic analysis for known mtDNA mutations associated with major mitochondrial diseases such as MELAS, MERRF and NARP (neuropathy, ataxia, retinitis pigmentosa) with a negative result. Therefore, we can hypothesize that deleterious variants of nuclear genes encoding mitochondria-related proteins, leading to mitochondrial dysfunction, in combination with the mitochondrial 9 bp deletion, contribute to the observed heterogeneity.

The effect of the MIC9D polymorphism on cognitive impairment may or may not depend on additional factors and further studies are needed. Due to its specific location, the MIC9D polymorphism can alter upstream and downstream gene expression and a potential effect on mtDNA replication and translation is suggested. Our data strengthen the hypothesis that there is a mitochondrial dysfunction component in cognitive impairment and provide a further rationale for the use of the MIC9D polymorphism for targeted screening in families with cognitive impairment, as well as for studies of future mitochondria-directed gene therapies in this condition, with the aim of improving the quality of life of the patient and their family.

Based on our findings, we propose the MIC9D polymorphism as a disease risk factor in the Caucasian population.

We cannot dismiss the high variability in our sample of patients with cognitive disorders. It is relevant to underline that we found an MIC9D polymorphism in six Sicilian patients with more complicated clinical presentations, but we believe that further studies are needed in a larger sample of individuals as well as in Caucasian/European populations to understand whether this polymorphism could have a role in cognitive impairment with concurrent involvement of neuromuscular and behavioral disorders.

## 4. Materials and Methods

### 4.1. Patient Population

This study included a convenience sample of 154 patients (99 males and 55 females; age ranging from 2 to 69 years) with cognitive deficits (intellectual disability, cognitive decline, learning disability, developmental delay) recruited at the Neuromuscular Unit of the Oasi Research Institute.

The evaluation of neuromuscular signs was made through a detailed clinical analysis during neurological examination.

The emotional–behavioral manifestations have been defined, according to age, frequency and symptoms with which they manifest, in accordance with DSM-5 (Diagnostic and Statistical Manual of Mental Disorders, 5th ed.) [23] and ICD-10 (WHO, 1992) [24]. To do this, in addition to the anamnestic data, clinical behavioral observation, behavioral checklists, interviews and/or questionnaires to parents or caregivers or patients were used where possible.

To explore the frequency of the MIC9D polymorphism in a Sicilian population, 147 control individuals underwent genetic analysis. Informed consent for study participation was obtained from all patients or, when necessary, from their relatives. The study was conducted in accordance with the Declaration of Helsinki of 1964 and its later amendments. The protocol was approved by the local ethics committee (Comitato Etico IRCCS Sicilia-Oasi Maria SS) on 5 April 2022 (2022/04/05/CE-IRCCS-OASI/52).

### 4.2. Genetic Analysis

After informed consent was given, the total genomic DNA of the patients was isolated from peripheral blood using standard protocols.

A pair of primers (5′-ACGAGTACACCGACTACGGC-3′ and 5′-TGGGTGGTTGGTGTAAATGA-3′) was designed by the software Vector NTI Advance 10.3.0 (Informax, Frederick, MD, USA) to amplify the CCCCCTCTA repeat in the non-coding region of the mtDNA between the COII and lysine tRNA genes. In order to analyze the PCR products, the forward primer was tagged with 5′ FAM fluorescence dye. PCR reactions were carried out using the manufacturer’s instructions. Briefly, 50 µL reaction volumes containing 200 ng genomic DNA, 1X PCR reaction buffer, 0.2 mM of each dNTP, 1 µM of each primer and Taq DNA polymerase (Roche, Mannheim, Germany) were used. PCR cycling conditions used for the amplification included an initial denaturation step at 94 °C for 5 min, followed by 30 cycles of 30 s at 94 °C, 45 s at 61 °C, 2 min at 72 °C and a final extension step at 72 °C for 5 min.

For genotyping, the PCR product was mixed with GS LIZ500TM size standard and Hi-Di formamide and analyzed on an ABI310 automated DNA sequencer (Applied Biosystems, Foster City, CA, USA). The raw data were further analyzed using GeneMapper software 6.0 (Applied Biosystems).

For anomalous profiles, the 9 bp deletion was confirmed with sequencing as previously described [11]. Briefly, PCR products were sequenced using a BigDye Terminator Cycle Sequencing Kit purified with a DyeEx 2.0 Spin Kit (Qiagen, Hilden, Germany) and analyzed on an ABI310 automated DNA sequencer (Applied Biosystems, Foster City, CA, USA). Patient sequence data were aligned for comparison with the corresponding wild-type sequence (NC_012920.1).

The patients with MIC9D polymorphism were screened for known common mtDNA mutations associated with major mitochondrial diseases such as MELAS, MERRF and NARP (Neuropathy, Ataxia, Retinitis Pigmentosa) by RFLP analysis. PCR products were digested and separated on a non-denaturing polyacrylamide gel and subjected to autoradiography.

### 4.3. Statistical Analysis

We verified if there was an association between cognitive disorders and the presence of MIC9D polymorphism. In addition, in a group of patients with cognitive disorders, we tested the association between MIC9D polymorphism and neuromuscular and behavioral disorders. For the categorical variables, we used Fisher’s exact test. For all analyses, the significance threshold was achieved with *p* < 0.05.

These statistical analyses were performed in Social Science Statistics (2024) software (https://www.socscistatistics.com/tests/fisher/default2.aspx, accessed on 10 July 2024).

## Figures and Tables

**Table 1:** Analysis of the 9 bp repeats in subjects with or without cognitive deficits.

	With Cognitive Deficits		Without Cognitive Deficits	
One repeat (deletion)	6	(3.9%)	0	(0.0%)
Two repeats	148	(96.1%)	147	(100.0%)
	(*n* = 154)		(*n* = 147)	

**Table 2:** The baseline and clinical characteristics of patients with cognitive deficits.

Characteristics		*n* = 154	Frequencies (%)	
Gender				
	Male	99	64.29	%
	Female	55	35.71	%
Neuromuscular disorders				
	Muscle hypotonia	53	34.42	%
	Motor coordination disorder	15	9.74	%
	Muscle pain	3	1.95	%
Cognitive deficits				
	Intellectual disability/global developmental delay	130	84.42	%
	Language disorder	47	30.52	%
	Specific learning disorder	13	8.44	%
	Developmental coordination disorder	6	3.90	%
	Dementia	3	1.95	%
Behavioral disorders				
	Unspecified mood disorder	8	5.19	%
	Unspecified anxiety disorder	6	3.90	%
	Sleep disorder, unspecified	6	3.90	%
	Oppositionism	6	3.90	%
	Aggressiveness	5	3.25	%
	Attention-deficit/hyperactivity disorder	4	2.60	%
	Provocative attitudes	3	1.95	%
	Self-harm	3	1.95	%
	Obsessive compulsive disorder	2	1.30	%
	Unspecified personality disorder	2	1.30	%

**Table 3:** Clinical features in patients with 9 bp deletion.

			Neuromuscular Disorders			Behavioral Disorders			
Patient	Sex	Age	Hypotonia	Hypotrophy	Motor Coordination Disorders	Oppositionism	Aggressiveness	Provocative Attitudes	Self-Harm
1	M	8.6	+	+	-	+	+	+	-
2	F	7.3	+	-	-	+	-	-	-
3	M	7.2	+	-	-	+	-	-	-
4	F	13.6	-	-	+	-	+	+	+
5	M	3.0	+	+	-	-	-	-	-
6	M	4.3	+	-	-	+	+	-	+

**Table 4:** Analysis of the 9 bp repeats in patients with cognitive deficits with or without behavioral disorders.

		Behavioral Disorders		Without Behavioral Disorders	
One repeat (deletion)	(*n* = 6)	5	(83.3%)	1	(16.7%)
Two repeats	(*n* = 148)	27	(18.2%)	121	(81.8%)

**Table 5:** Analysis of the 9 bp repeats in patients with cognitive deficits with or without neuromuscular disorders.

		With Neuromuscular Disorders		Without Neuromuscular Disorders	
One repeat (deletion)	(*n* = 6)	6	(100.0%)	0	(0.0%)
Two repeats	(*n* = 148)	61	(41.2%)	87	(58.8%)

## Data Availability

Data are available from the corresponding author upon a reasonable request.
